# Annotations

**Published:** 1898-05-28

**Authors:** 


### Annotations.

### Statutory Holidays.

If we are to believe the reports from Melbourne the dwellers in that good town are tasting the sweets of democracy in the form of sour milk. What democracy has to do with milk is not at first sight quite clear, but the thing works out in this way. Melbourne is a highly democratic place, and the workmen having decided to have a half-holiday every Wednesday, they are not content to take it, but must needs have it enforced by law, so Melbourne has its statutory holidays on Sunday and Wednesday afternoons. Now the law on this subject applies also to dairymen and milk dealers, with this interesting result, that while labour lounges in compulsory idleness (for labour laws must not be broken), the milk goes sour. Hence all sorts of woes. A medical officer of the Children's Hospital in Melbourne says that "the prohibition of the supply of fresh milk on Wednesdays and Sundays is a scandal and a disgrace to a civilised community." Nine-tenths of the cases of infantile diarrhœa are, he says, attributable to sour milk (in which no doubt he is right), and innumerable cases are noticed to take their rise from Monday and Thursday, the days following the deliveries of the stale milk. Another medical man says that "most certainly the holiday is the cause of great infantile mortality." All of this may be quite true, and as we do not know whether the dairymen of Melbourne are so law-abiding as to stand idly by while their milk goes sour according to statute, we can say nothing as to the facts of the case. We would point out, however, that we in England are hardly yet awake to the possibilities of the noble art of politics; and when we remember how in Adelaide the hospital affairs were bandied about for party purposes we can hardly be blind to the possibility that the high infantile mortality of Melbourne has been seized hold of by some astute politician, and has been turned to prove the baseness, the heartless cruelty, and all the rest of it, of whichever party happened to favour the statutory half-holiday! Still, if the facts are as related, we are sorry for the babies, and we may add also for the cows.

### Medical Ethics.

The *British Medical Journal* is a serious paper, so it puts its jokes in small type at the end. Nevertheless they are sometimes fairly funny. We extract the following conundrum and its answer from the "Queries" column of last week's issue:—

> "A. is a member of a workman's club. B. is A.'s family's medical attendant. C. is medical officer of A.'s club. When A. became ill he sent for his club doctor, C., to attend him, but after a time he left C., and sent for B. to attend him, who then took up the case. What ethical conditions are involved?"

After entering into various matters of more or less interest, the editorial comment ends as follows:—

> "When A. sent for B. strict ethics would require him to refuse attendance until he was satisfied A. had explained to C. his intention of consulting B.; but in this case one friendly line from B. to C. would render the matter simple."

We quite agree that the "friendly line" is a good way out of the difficulty, but what we are interested in is the *British Medical Journal's* notions as to what strict ethics require. Let us imagine for a moment what would be the condition of affairs if the whole medical profession were to feel bound by the rules of "strict ethics," as defined by the *Journal*. Mr. A. wishes to try a new doctor. The first thing, of course, is to get rid of the old one. Perhaps he pays him what he owes; but, at any rate, the doctor ceases to attend. All this is simple enough. But when he goes to ask Mr. B. to come, Mr. B. declines, because, forsooth, "strict ethics would require him to refuse attendance until he was satisfied that Mr. A. had explained to" his old doctor that he was going to consult him! And so he may go all round the town, but if all the doctors should happen to be sticklers for "strict ethics," which heaven forefend! each of them will "refuse attendance" until he is, in some way or other, satisfied that the unfortunate man had explained his intention of consulting him to his predecessor. But surely all this setting up of artificial and arbitrary rules of conduct is very petty and ridiculous. If a man chooses to change his doctor, what is there to prevent him doing so? and why should anybody feel aggrieved so long as it is done openly and honestly? We can understand a medical man feeling sore, and anyone can understand the meanness of the action, when a patient quietly runs up to Harley Street and gets an "independent opinion," while his own doctor is actually attending; but if a man has given his doctor up, we fail to see what there is to prevent him going to whom he pleases. He has the world before him, and can make his choice, and we entirely dissent from the doctrine that the medical man whom he then consults is required by "strict ethics" to "refuse attendance" until satisfied that the would-be patient has explained to his old doctor that he was going to consult the one on whom his choice has fallen. It is the insistence upon fanciful rules like this, which, by the bye, have no foundation in the practical work of life, that leads people to sneer at what they call "medical etiquette."

### Stuffy Houses.

The subject of the outdoor treatment of phthisis, which is dealt with in another column, surely has its lessons for the healthy. The admitted advantage of an outdoor life in many morbid conditions, and notably in consumption, seems to point to the conclusion that there is something definitely injurious in the indoor life which is now the common mode of existence among civilised people. It is a striking and startling thing that the mere removal of a patient into the open air should lower his fever, should remove his night sweats, and take away his hectic, and it is difficult to avoid the conclusion that if these symptoms are removed by the purity of the air outside they must have been largely caused by the impurity of the air within the house. Nor have we any right to assume that it is the consumptive only who suffers. Doubtless the healthy struggle against and overcome evil influences before which those who are tuberculous succumb, but that is not to say that in the struggle we do not suffer, and, indeed, the facts recently brought forward are sufficient to show that the stuffy life of warmth and comfort which civilised man now "enjoys" is bad for the health even of the healthiest. We make our windows fit, we pad our doors, we shiver at a draught, we surround ourselves with woollen curtains, dusty carpets, and fluffy luxurious upholstery; we breathe the same air over and over again, and then we wonder that we are not strong and vigorous. The fact is we are daily using up the exuberant vitality with which nature has provided us in struggling against artificial conditions. How powerful for evil, how deteriorating these conditions are, is shown by the fact that their mere removal gives back to the consumptive that vitality which enables him to overcome the seeds of disease within him. Fresh air is not a thing to be taken in little doses once a day, but a thing to live on.

